# An outdoor dual-task study on cognitive-motor interference during exoskeleton-assisted walking

**DOI:** 10.3389/fpsyg.2025.1583142

**Published:** 2025-06-16

**Authors:** Norman Riedel, Giorgos Marinou, Katja Mombaur, Barbara Deml

**Affiliations:** ^1^Institute of Human and Industrial Engineering, Karlsruhe Institute of Technology, Karlsruhe, Germany; ^2^Institute of Computer Engineering, Heidelberg University, Heidelberg, Germany; ^3^Institute for Anthropomatics and Robotics, BioRobotics Lab, Karlsruhe Institute of Technology, Karlsruhe, Germany; ^4^Systems Design Engineering and the Department of Mechanical and Mechatronics Engineering, University of Waterloo, Waterloo, ON, Canada

**Keywords:** cognitive-motor interference, lower-limb exoskeleton, human-exoskeleton interaction, attentional resources, cognitive fit, familiarization, dual-task walking

## Abstract

**Introduction:**

Controlling a powered lower-limb exoskeleton may increase the demand for cognitive resources due to mechanical constraints and assisting torques that disrupt natural movement.

**Method:**

This study employed a dual-task paradigm to investigate cognitive-motor interferences and short-term familiarization effects in an outdoor walking experiment with twenty healthy adults using a powered lower-limb exoskeleton.

**Results and discussion:**

In contrast to unassisted walking, performing a serial subtraction task during exoskeleton-assisted walking led to a slight increase in gait velocity and a reduction in stride time variability. This suggests that externalizing attention may facilitate the coordination with external rhythmic cues provided by the exoskeleton. Concurrently, cognitive performance, measured by correct response rates, declined during exoskeleton-assisted walking, indicating a *posture-first* strategy. Short-term familiarization during exoskeleton-assisted walking reduced perceived workload and improved cognitive performance, yet cognitive performance remained lower than in both the seated control condition and unassisted walking. This suggests that walking with the exoskeleton continues to require significant attentional resources. These findings emphasize the necessity of evaluating the cognitive fit of exoskeletons to ensure safe human-exoskeleton interaction.

## 1 Introduction

In recent years, the integration of lower-limb exoskeletons as walking assistance devices has opened new avenues for enhancing mobility. Given the demographic shift toward an aging population, exoskeletons offer a promising solution to help the elderly maintain autonomy and independence in their everyday life (Kapsalyamov et al., 2019). Although lower-limb exoskeletons are currently available for use in controlled clinical and laboratory settings, their application in real-world environments remains a work in progress (Sawicki et al., 2020). Current performance evaluations of lower-limb exoskeletons predominantly focus on biomechanical and physiological parameters, often neglecting the cognitive aspects of the interaction (Pinto-Fernandez et al., 2020). However, Stirling et al. (2020) emphasize the importance of evaluating the cognitive fit in addition to the static and dynamic fit of an exoskeleton to ensure that the cognitive abilities necessary for other relevant tasks are fully available even when using an exoskeleton. Therefore, in addition to the technical challenges that have been identified (e.g., Shi et al., 2019; Kapsalyamov et al., 2019), the cognitive-motor interferences that arise from human-exoskeleton interaction remain a critical area of investigation.

Dual-task walking paradigms are an established approach to investigate cognitive-motor interference, particularly in clinical and epidemiological research (Al-Yahya et al., 2011; Beurskens and Bock, 2012; Kelly et al., 2012). In dual-task scenarios, limited cognitive resources can lead to performance declines in one or both tasks, influenced by individual characteristics and task complexity (Yogev-Seligmann et al., 2012). For instance, healthy young adults can allocate attention to a secondary task while maintaining a stable gait due to their high postural reserve. The postural reserve is defined as the capacity to respond optimally to a postural threat (Yogev-Seligmann et al., 2012). In contrast, elderly individuals, due to age-related cognitive decline, need to allocate more attentional resources to the motor task (Yogev-Seligmann et al., 2012). A motor task with increased balance control requirements, such as walking with an exoskeleton, may be perceived as a postural threat. Such complex motor tasks may cause attentional resources to be allocated to the motor task at the expense of the cognitive task, even in healthy young adults (Bequette et al., 2020; Hinton et al., 2020; Kao and Pierro, 2022; Mersmann et al., 2013; Reiser et al., 2019; Riedel et al., 2024).

To assess cognitive-motor interference and task prioritization, it is necessary to quantify both cognitive performance and motor performance using a multimodal approach. Cognitive performance is often measured using behavioral parameters like secondary task performance, whereas motor performance in dual-task walking studies is mainly evaluated through various gait parameters. Gait velocity is the most common parameter for assessing motor performance. Individuals generally slow down when a secondary task is introduced, especially if it involves complex neural networks that are interconnected with motor control, like mental tracking (Al-Yahya et al., 2011). Another key indicator of motor control is stride-to-stride variability (Hausdorff, 2005). Low variability indicates the reliance on automatic processes, while high variability suggests the engagement of attentional resources in motor control.

Powered lower-limb exoskeletons typically feature rigid segments and active joints, which can restrict the range of motion and modify mass and inertia distribution (Jin et al., 2017). It has been hypothesized that walking with an exoskeleton requires increased attentional resources to generate the appropriate muscle recruitment patterns necessary for maintaining a stable gait (Andrade et al., 2024). Additionally, imprecise application of active support can disrupt natural motion execution, requiring simultaneous control of both the device and the user's own motion, thereby increasing cognitive load (Clark, 2015) and physical load in terms of fatigue and metabolic costs (Stirling et al., 2020). Bequette et al. (2020) found that wearing a powered lower-limb exoskeleton led to slower reaction times in a visual search task for some participants. Moreover, the perceived workload, as measured by the NASA-TLX, was significantly higher during both powered and unpowered walking compared to walking without the exoskeleton.

**Figure 1:**
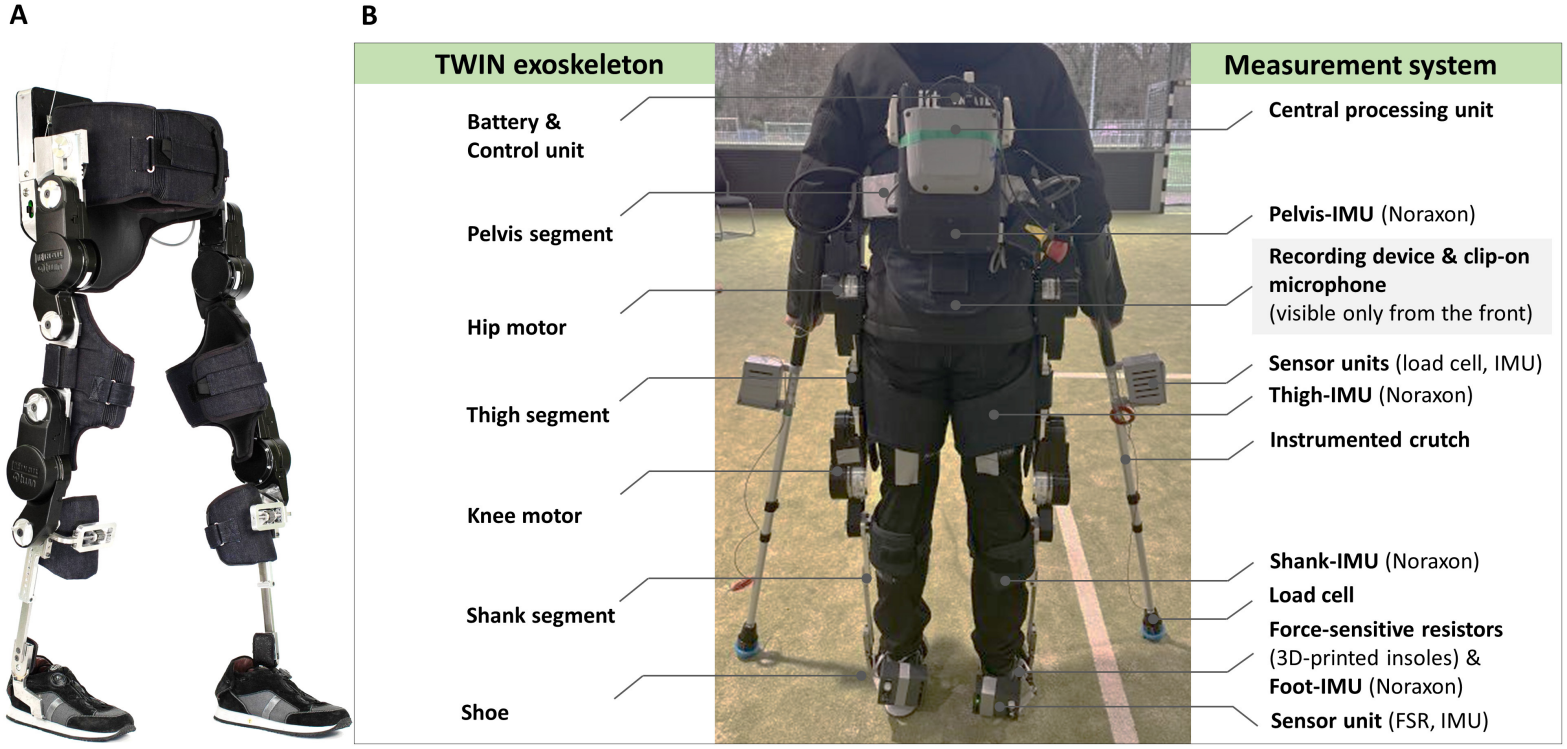
**(A)** TWIN lower-limb exoskeleton (adapted from Semprini et al., 2022), **(B)** setup of the TWIN exoskeleton and the measurement system. FSR, force sensitive resistor; IMU, inertial measurement unit.

This study employed the powered lower-limb exoskeleton TWIN (Laffranchi et al., 2021) (see Figure 1) to investigate cognitive-motor interference and short-term familiarization effects in an outdoor dual-task walking experiment with a cohort of healthy young adults. The TWIN utilizes position control with predefined gait trajectories, requiring users to synchronize their movements with the exoskeleton. The *primary hypothesis* (*H1*) of this study postulates that cognitive-motor interference increases during exoskeleton-assisted walking compared to unassisted walking, resulting in decreased cognitive performance, motor performance, or both. Additionally, it was hypothesized that perceived mental and physical workload increase. However, research on adaptation to exoskeleton walking has shown improved muscle recruitment patterns (Gordon and Ferris, 2007; Jacobs et al., 2018) and significant reductions in energy costs (Poggensee and Collins, 2021). Clark (2015) provides evidence that excessive physical exertion requires more cognitive resources. This suggests that greater alignment with the exoskeleton's assistance may result in a reduction in cognitive demands. The *secondary hypothesis* (*H2*) predicted that short-term, within-session familiarization during exoskeleton-assisted walking improves both cognitive and motor performance and decreases perceived workload. The investigation of familiarization with lower-limb exoskeletons is extended through a complementary study by Marinou et al. (manuscript in preparation), where biomechanical metrics are established as familiarization indicators, quantifying familiarization through single and dual task conditions by systematically measuring variables such as stride duration, crutch ground reaction forces and foot center of pressure. Together, these studies enhance the understanding of familiarization by integrating both cognitive and biomechanical insights, thereby advancing research on the broader impacts of exoskeleton use on human performance.

## 2 Materials and methods

### 2.1 Participants

Twenty healthy young adults (age: 25.3 ± 4.1 years; stature: 1.73 ± 0.10 m; body mass: 69 ± 12.1 kg; 10 female, 10 male) participated in the study. The participants were students at the Karlsruhe Institute of Technology (KIT). Participants had to be between 1.55 and 1.90 m tall and weigh < 100 kg so that the exoskeleton could be adjusted accordingly. This research was conducted in accordance with the American Psychological Association Code of Ethics and received approval from the KIT-Ethics Committee. Prior to participation, all subjects provided written informed consent.

### 2.2 Apparatus

Data collection took place at the KIT sports facilities on a 25 m track in a covered open-air hall. During the walking sessions, the powered lower-limb exoskeleton TWIN was used, along with a measurement system that included various inertial, force, and pressure sensors (see Figure 1).

#### 2.2.1 TWIN exoskeleton

The TWIN was developed at the IIT-INAIL Rehab Technologies Lab with the primary objective of assisting the ambulation of spinal cord injury patients. It was designed to operate in full position control, generating predefined movement trajectories that can be modified based on gait parameter inputs by the operator (Vassallo et al., 2020). Based on Vassallo et al. (2020), a predetermined trajectory (T3) is incorporated in the TWIN's high-level control, chosen based on the merits of joint angle minimization during walking as to maximize user comfort, and heel-strike phase accentuation to avoid stumbling during gait, thus increasing system stability. The modular structure of the TWIN comprises four actuated joints at the hips and knees and five rigid components at the pelvis, as well as right and left components for the thighs and shanks with padded braces. The rigid components are available in different sizes, allowing for individual anthropometrical fitting of the exoskeleton. The controller and batteries are located at the lower back. While the exoskeleton provides rigid support, the hip and knee joints allow for minor ranges of motion for internal and external rotations. Additionally, the ankle joint incorporates a variable elastic element to accommodate variations in dorsiflexion and extension. Consequently, despite the predefined joint trajectories, the user can introduce within-step variability.

The gait parameters for the trajectory generation can be controlled through a mobile device using a custom app. TWIN can operate in two walking modes: manual and automatic. In manual mode, an external person triggers each step via the app. In automatic mode, an inertial measurement unit (IMU) located at the back element of the TWIN initiates a step sequence upon detecting a slight forward shift of the trunk. The sequence continues until the user returns to an upright position, with the final step safely bringing the feet together. Since the range of motion is primarily restricted to the sagittal plane (flexion-extension), this can challenge both static and dynamic balance, necessitating the use of forearm crutches.

#### 2.2.2 Measurement system

Kinematics were assessed at 200 Hz using a Noraxon Ultium Motion IMU system with MyoResearch 3.20.40 software, incorporating seven IMUs at the feet, shanks, thighs, and pelvis (Noraxon U.S.A. Inc., Scottsdale, AZ). Following the instructions provided by Noraxon, lower-limb sensors were affixed to a Velcro strap that was fastened around the corresponding body segment. The pelvis sensor was attached directly to the skin via an adhesive. In order to capture the participant's responses in the secondary task for the analysis of the cognitive performance, a recording device (Sony, model: ICDUX570) with a clip-on microphone (Phillips, model: LFH9173/00) was used.

The crutches used in this study were instrumented with load cells and IMUs. Additionally, force-sensitive resistors within a 3D-printed insole were placed in the exoskeleton shoes (Marinou et al., 2025). The data collected from these sensors helped generate the biomechanical metrics used as familiarization indicators in the parallel study by Marinou et al. (manuscript in preparation).

### 2.3 Procedure

The study involved two separate appointments. During the initial preparation appointment, participants provided written informed consent and anthropometric measurements were taken to ensure the exoskeleton was individually fitted. Participants received detailed instructions on how to use the exoskeleton and crutches before taking a maximum of five steps with the exoskeleton in manual mode, limiting familiarization effects prior to data collection.

**Figure 2:**
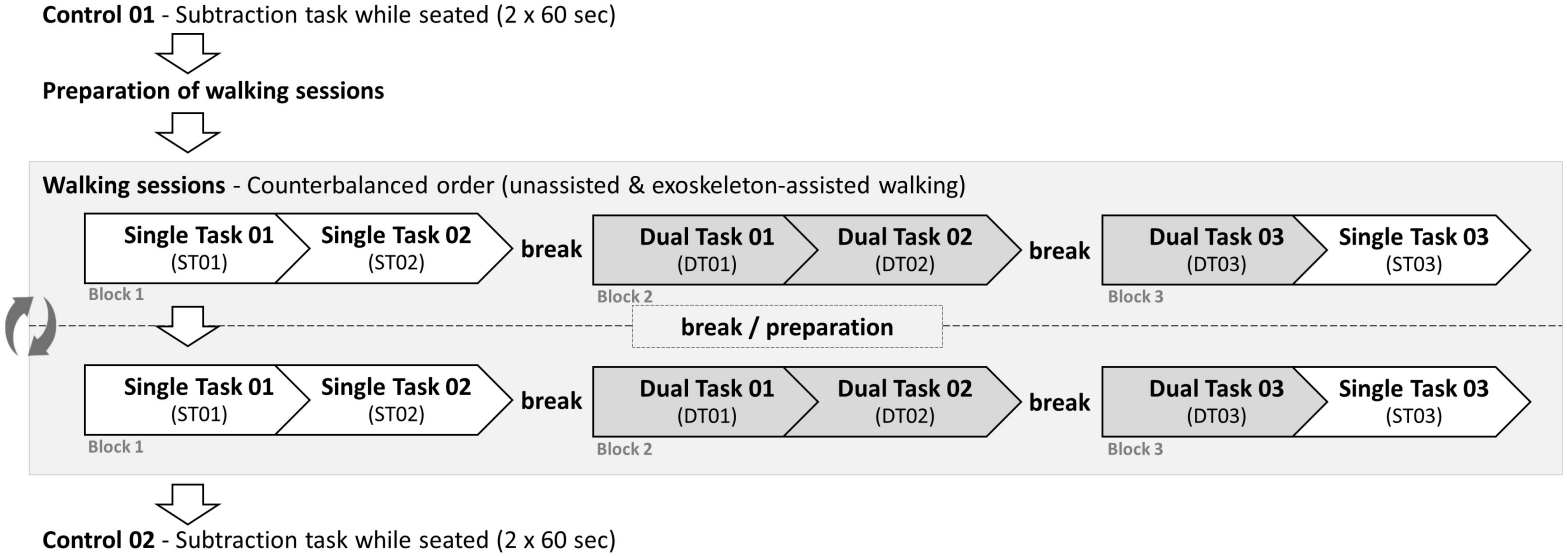
Experimental procedure at the second appointment.

Data collection during the second appointment took place at least 14 days after the initial appointment. The exoskeleton and the instrumented crutches were preadjusted based on the measured individual anthropometric parameters. The procedure at the second appointment is illustrated schematically in Figure 2.

The cognitive task was initially performed while seated to allow for familiarization with the task. Participants performed a serial subtraction task in which they were given a random three-digit number between 301 and 999 and instructed to subtract 7 repeatedly as quickly and accurately as possible. Participants performed two trials, each lasting 60 s (Control 01). After each trial, participants rated their perceived mental and physical workload using the respective NASA-TLX subscales (Hart and Staveland, 1988).

Noraxon IMUs were attached and calibrated dynamically in accordance with the walking calibration instructions. First, subjects stood still for 2.5 s with their arms at their sides and their feet close to shoulder-width apart. They then walked for 15 s at a self-selected speed, made a 180° turn, walked back to the starting position, and stood still another 2.5 s in the same initial posture. After the calibration, participants engaged in two walking sessions, an unassisted walking session and an exoskeleton-assisted walking session, in a counterbalanced order. Both sessions comprised of three blocks with two walking trials of 25 m each. After the first walking trial in each block, participants turned 180° and walked back after an obligatory short break (~2 min), followed by an obligatory longer break (~5 min) between blocks. Both walking trials of the first block were carried out without a cognitive task, i.e. single-task walking (ST01 and ST02). In the second block, the participants performed the subtraction task in both walking trials, i.e. dual-task walking (DT01 and DT02). In the third block, the first walking trial included the subtraction task, whereas the second walking trial did not (DT03 and ST03). In the short and long breaks between trials, the participants again rated their perceived mental and physical workload.

During the unassisted walking session, only the lower-limb IMUs and audio recorder were utilized. Participants walked at their preferred walking speed at an average of 1.31 ± 0.08 m/s (4.72 ± 0.29 km/h). In contrast, during the exoskeleton-assisted walking session, the entire measurement system was employed. Participants were first fitted with the exoskeleton while seated, including replacing their shoes with the exoskeleton's integrated shoes. Subsequently, the foot IMUs were transferred to the TWIN shoes. This was followed by a recapitulation of the instructions for using the exoskeleton in automatic mode. Finally, the pressure sensors, IMUs on the crutches, and load cells were calibrated. The average walking speed across exoskeleton conditions was 0.10 ± 0.01 m/s (0.36 ± 0.03 km/h). Several factors influence exoskeleton-assisted walking speed, with the control strategy being a primary contributor, which allows for pre-programmed step parameters. This includes parameters such as step velocity and inter-step timing, while anthropometric scaling further influences timing by adjusting step length based on the user's segment lengths. These factors likely contributed to reduced walking speeds, as participants needed to synchronize with the exoskeleton's movements, although they retained some control over step timing by leaning back.

After the two walking sessions, participants performed the subtraction task while seated to assess cognitive performance without the motor task (Control 02). As at the beginning, there were two 60-s trials. The second appointment took a maximum of 180 min.

### 2.4 Data processing

To quantify cognitive performance, the correct response rates (CRR) were calculated. According to Galletly and Brauer (2005), this is determined by multiplying the response rate (subtractions per second) by the accuracy (percentage of correct subtractions). During the exoskeleton-assisted walking trials, data were collected from a 60-s segment following an initial 60 s of walking. For unassisted walking trials, the entire duration was used.

To evaluate the motor performance, stride time, double support time and gait velocity were calculated using MATLAB R2023a (The MathWorks, Natick, MA, United States). Stride time is the duration of one complete gait cycle, from initial contact of one foot to its subsequent contact. Double support time is the phase within the gait cycle when both feet are simultaneously in contact with the ground. For stride time and double support time, the mean values and coefficients of variation (CV) were determined. The CV were calculated by dividing the standard deviation by the mean and multiplying by 100.

**Figure 3:**
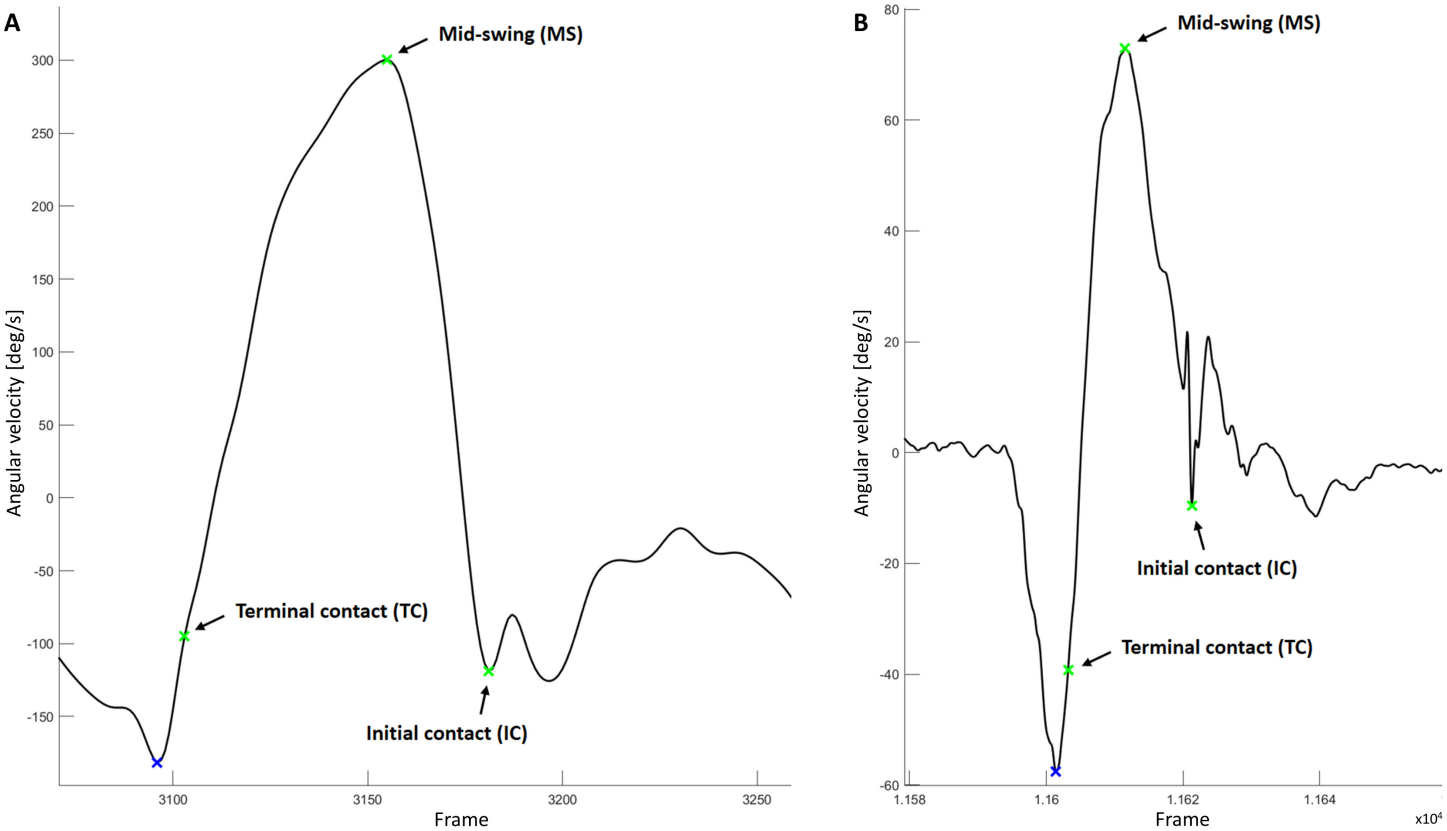
Gait event detection with the Dual Minima Method based on angular velocity of the shank-mounted IMU. Exemplary extraction of a single step of participant 01 during **(A)** unassisted walking and **(B)** exoskeleton-assisted walking.

Gait event detection was conducted using the Dual Minima Method, which relies on the angular velocity measured by gyroscopes mounted on the shank (Aminian et al., 2002; Bötzel et al., 2016; Greene et al., 2010; Salarian et al., 2004). After applying a 20 Hz, 4th-order low-pass Butterworth filter to the angular velocity data, local maxima were identified as mid-swing (MS). Initially, the preceding and following local minima below zero were marked as initial contact (IC) and terminal contact (TC), respectively. While IC detection was accurate, TC was found to occur later than initially detected (Bötzel et al., 2016). Following Bötzel et al. (2016), TC was defined as the midpoint between the minimum and the zero-crossing (see Figure 3). Since symmetry was assumed, the stride time was calculated based on the right side. For each walking trial, the first and last two strides were excluded from the analysis to account for acceleration and deceleration effects. The analysis was based on an average of 23 strides performed with the exoskeleton and 15 strides without it.

Gait velocity was calculated by dividing the distance walked by the time taken. The distance was assessed based on the pelvis IMU trajectory calculated via double integration in Noraxon MyoResearch. The magnitude of the resulting trajectory was calculated in the antero-posterior and medio-lateral directions. Due to the progressive accelerometer drift of the Noraxon IMUs during the lengthy exoskeleton-assisted walking trials, the gait velocity data were extracted only for the first single- (ST01) and dual-task (DT01) trials.

### 2.5 Statistical analysis

**Figure 4:**
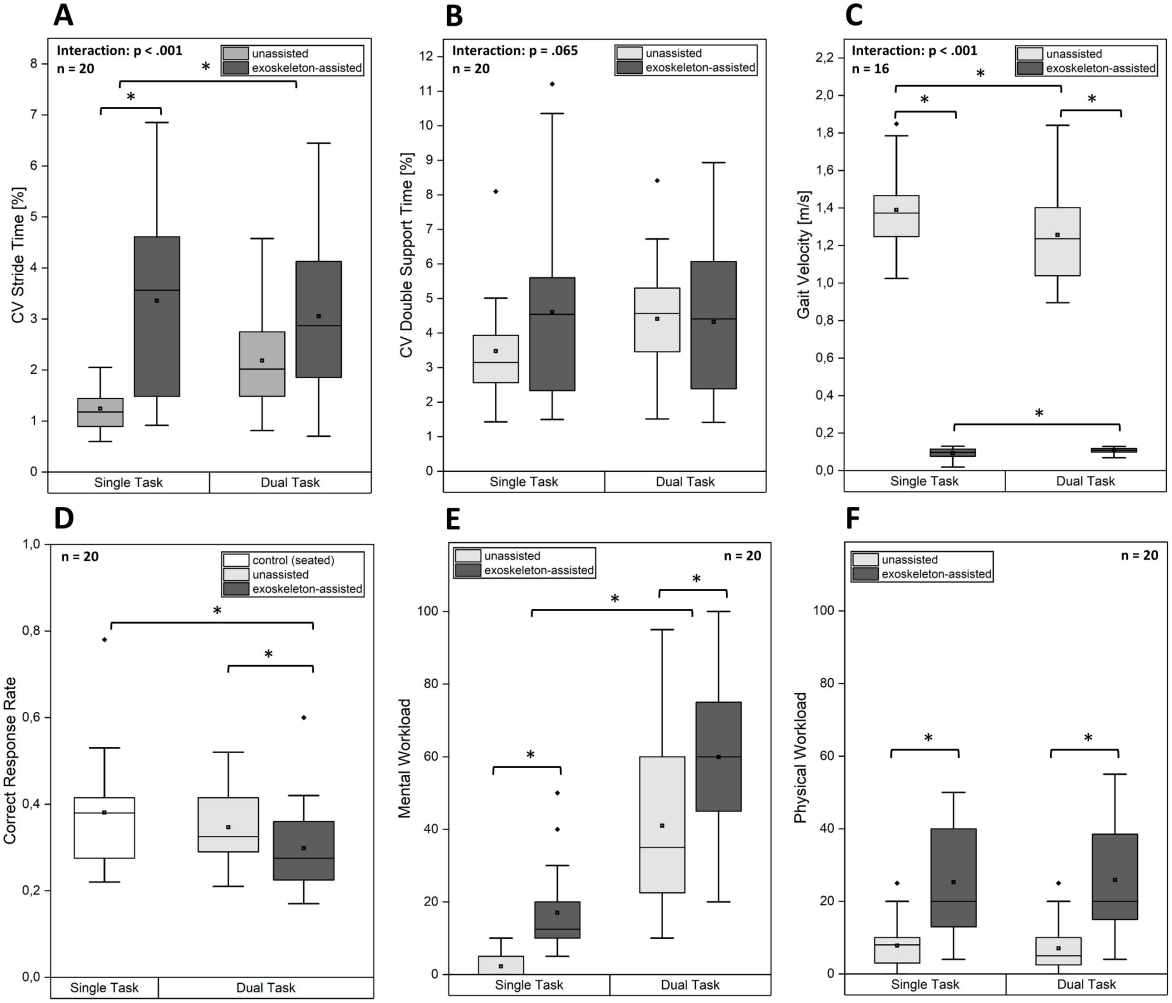
Boxplots depicting the **(A)** coefficient of variation (CV) of stride time, **(B)** CV of double support time, **(C)** gait velocity, **(D)** correct response rate, **(E)** mental workload and **(F)** physical workload across different task conditions and walking conditions. The median (horizontal line within the box), the mean (black squares), the interquartile range (box), and the data spread including potential outliers (dots) are shown for each condition. Asterisks (*) indicate statistically significant differences between conditions.

**Figure 5:**
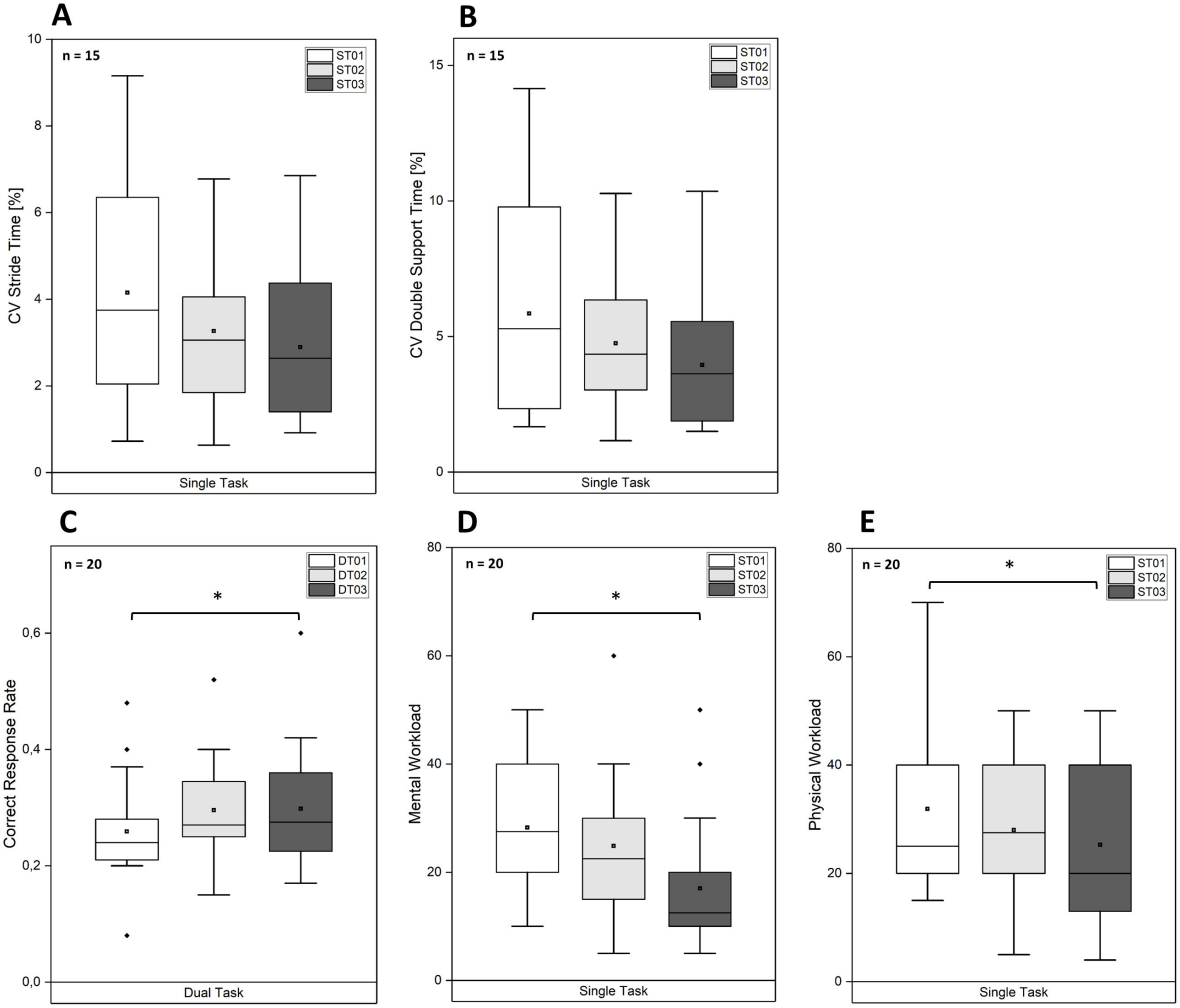
Boxplots depicting the **(A)** coefficient of variation (CV) of stride time, **(B)** CV of double support time, **(C)** correct response rate, **(D)** mental workload and **(E)** physical workload for exoskeleton-assisted walking across the three single-task trials. Note that correct response rates were only assessed for the dual-task trials. The median (horizontal line within the box), the mean (black squares), the interquartile range (box), and the data spread including potential outliers (dots) are shown for each condition. Asterisks (*) indicate statistically significant differences between conditions.

Normality of the data was assessed using the Kolmogorov-Smirnov test. Given that repeated measures ANOVAs (rmANOVA) are robust to deviations from normality when the sphericity assumption is satisfied (Blanca et al., 2023; Schmider et al., 2010), parametric models were employed. If both assumptions were violated, non-parametric models were used instead. Sphericity was evaluated with the Mauchly test, and when violated, the Greenhouse-Geisser correction was applied. All statistical tests were conducted with a significance level of α = 0.05, with Bonferroni corrections applied to *post-hoc* pairwise comparisons. The effect sizes are reported as partial eta squared ($\eta_{p}^{2}$). Measurement errors in certain trials led to data loss, reducing the sample size for some parameters. The specific sample sizes are indicated in the results section through the reported degrees of freedom and are annotated in each plot shown in Figures 4, 5. The statistical analyses were conducted using SPSS Statistics 29.0 (IBM, Armonk, NY, USA).

To test the first hypothesis, 2 × 2-rmANOVAs were performed for stride time variability, double support time variability, gait velocity, and perceived mental and physical workload. The analyses included two within-subject factors: *Walking Condition* (Unassisted walking, exoskeleton-assisted walking) and *Task Condition* (Single-task, dual-task). Data from the third block (DT03, ST03) were used for the analysis, as the first two blocks functioned as a familiarization phase (see Figure 2). However, gait velocity analysis included data from ST01 and DT01, as described above. A 1 × 3-rmANOVA was conducted on CRR, including the dual-task conditions and the second seated control condition (Control 02) as levels.

To test the second hypothesis, 1 × 3-rmANOVAs or non-parametric Friedman tests were conducted on the three single-task trials during exoskeleton-assisted walking, examining stride time variability, double support time variability, CRR, and perceived mental and physical workload. The analyses included *Time Condition* (ST01, ST02, ST03) as a within-subject factor. CRR was assessed exclusively during the dual-task trials (DT01, DT02, DT03).

## 3 Results

Table 1 presents the mean (SD) and median (IQR) for motor performance, cognitive performance, and perceived workload parameters in Block 3 (ST03, DT03) for both unassisted and exoskeleton-assisted walking, with corresponding boxplots depicted in Figure 4. Additionally, Table 2 presents the mean (SD) and median (IQR) of dependent variables for exoskeleton-assisted walking across the three single-task trials (ST01, ST02, ST03), with boxplots depicted in Figure 5.

**Table 1:** Mean (SD) and median (IQR) of motor performance, cognitive performance, and perceived workload parameters for unassisted and exoskeleton-assisted walking under single-task (ST03) and dual-task (DT03) conditions.

	**Unassisted walking**		**Exoskeleton-assisted walking**	
	**ST03**	**DT03**	**ST03**	**DT03**
**Motor performance**				
CV stride time (%)	1.2 (0.4) | 1.2 (0.9–1.5)	2.2 (0.9) | 2.0 (1.5–2.8)	3.4 (1.7) | 3.6 (1.5–4.6)	3.0 (1.6) | 2.9 (1.8–4.2)
CV double support time (%)	3.5 (1.5) | 3.1 (2.6–4.0)	4.4 (1.7) | 4.6 (3.4–5.4)	4.6 (2.6) | 4.5 (2.1–5.6)	4.3 (2.3) | 4.4 (2.3–6.5)
Gait velocity (m/s)	1.39 (0.21) | 1.37 (1.23–1.47)^1^	1.26 (0.25) | 1.24 (1.04–1.41)^1^	0.09 (0.03) | 0.10 (0.07–0.12)^1^	0.11 (0.02) | 0.11 (0.10–0.12)^1^
**Cognitive performance**				
Correct response rate	–	0.35 (0.09) | 0.33 (0.29–0.42)	–	0.30 (0.10) | 0.28 (0.22–0.36)
**Perceived workload**				
Mental workload	2.2 (3.3) | 0.0 (0.0–5.0)	41.0 (23.2) | 35.0 (21.3–60.0)	17.0 (12.3) | 12.5 (10.0–20.0)	59.9 (21.2) | 60.0 (42.5–75.0)
Physical workload	7.8 (6.4) | 8.0 (2.5–10.0)	7.1 (6.4) | 5.0 (2.3–10.0)	25.3 (16.2) | 20.0 (12.0–40.0)	25.9 (15.7) | 20.0 (15.0–39.3)

n = 20, 1: n = 16 and based on ST01, DT01.

CV, coefficient of variation; ST, single task; DT, dual task.

**Table 2:** Mean (SD) and median (IQR) of motor performance, cognitive performance, and perceived workload parameters for exoskeleton-assisted walking across the three single-task trials (ST01, ST02, ST03).

	**Exoskeleton-assisted walking**		
	**ST01**	**ST02**	**ST03**
**Motor performance**			
CV stride time (%)	4.2 (2.5) | 3.7 (2.0–6.4)^1^	3.3 (1.7) | 3.1 (1.8–4.1)^1^	2.9 (1.7) | 2.6 (1.4–4.4)^1^
CV double support time (%)	5.9 (3.7) | 5.3 (2.3–9.8)^1^	4.8 (2.4) | 4.3 (3.0–6.3)^1^	4.0 (2.3) | 3.6 (1.9–5.6)^1^
**Cognitive performance**			
Correct response rate	0.26 (0.08) | 0.24 (0.20–0.28)^2^	0.30 (0.08) | 0.27 (0.25–0.35)^2^	0.30 (0.10) | 0.28 (0.22–0.36)^2^
**Perceived workload**			
Mental workload	28.3 (12.2) | 27.5 (20.0–40.0)	24.9 (12.1) | 22.5 (15.0–30.0)	17.0 (12.0) | 12.5 (10.0–20.0)
Physical workload	31.9 (14.7) | 25.0 (20.0–40.0)	28.0 (11.4) | 27.5 (20.0–40.0)	25.3 (15.8) | 20.0 (12.0–40.0)

n = 20, 1: n = 15, 2: based on DT01, DT02, DT03.

CV, coefficient of variation; ST, single task.

### 3.1 Motor performance

The 2 × 2-rmANOVAs conducted to test H1 revealed that there was a statistically significant interaction between the effects of *Walking Condition* and *Task Condition* for stride time variability [*F*_(1, 19)_ = 15.30, *p* < 0.001, $\eta_{p}^{2}$ = 0.45] and gait velocity [*F*_(1, 19)_ = 34.98, *p* < 0.001, $\eta_{p}^{2}$ = 0.65]. Simple main effects tests indicated that stride time variability was significantly higher during exoskeleton-assisted walking than during unassisted walking in the single-task condition (*p* < 0.001). However, there were no significant differences during the dual-task condition (*p* = 0.062). Stride time variability during unassisted walking increased significantly from single-task to dual-task condition (*p* < 0.001), whereas it reduced on average during exoskeleton-assisted walking, however not significantly (*p* = 0.165). For gait velocity, simple main effects tests showed that participants reduced their speed from single-task to dual-task condition during unassisted walking (*p* < 0.001). In contrast, gait velocity during exoskeleton-assisted walking increased from single-task to dual-task condition (*p* = 0.018). The gait velocity during unassisted walking, under both single-task (*p* < 0.001) and dual-task conditions (*p* < 0.001), was significantly greater compared to exoskeleton-assisted walking. For double support time variability the rmANOVA showed no significant interaction effects [*F*_(1, 19)_ = 3.85, *p* = 0.065, $\eta_{p}^{2}$ = 0.17]. However, the statistical trend indicates a similar pattern observed for stride time variability. The main effects for *Walking Condition* [*F*_(1, 19)_ = 0.86, *p* = 0.365, $\eta_{p}^{2}$ = 0.04] and *Task Condition* [*F*_(1, 19)_ = 0.66, *p* = 0.426, $\eta_{p}^{2}$ = 0.03] were also not significant.

The 1 × 3-rmANOVAs conducted to test H2 revealed no significant effects of *Time Condition* for stride time variability [*F*_(1.30, 18.17)_ = 2.94, *p* = 0.095, $\eta_{p}^{2}$ = 0.17] and double support time variability [*F*_(1.29, 18.02)_ = 2.38, *p* = 0.136, $\eta_{p}^{2}$ = 0.15]. The statistical trends in stride time variability and double support time variability indicate a reduction in variability from the first to the third trial.

### 3.2 Cognitive performance

The 1 × 3-rmANOVA showed a significant effect for CRR [*F*_(2, 38)_ = 5.50, *p* < 0.001, $\eta_{p}^{2}$ = 0.38]. *Post-hoc* tests revealed that CRR was significantly lower during exoskeleton-assisted walking compared to both unassisted walking (*p* = 0.018) and the seated control condition (*p* < 0.001). No significant differences were found between unassisted walking and the seated control condition (*p* = 0.347).

A significant effect of *Time Condition* was observed [*F*_(2, 38)_ = 5.50, *p* = 0.008, $\eta_{p}^{2}$ = 0.23], indicating an increase in CRR from the first to the third trial during exoskeleton-assisted walking. *Post-hoc* analyses showed that CRR was significantly higher in the third trial compared to the first (*p* = 0.005), while no significant differences were observed between the first and second trial (*p* = 0.066) or between the second and third trial (*p* = 1.00).

### 3.3 Perceived workload

There was no significant interaction between *Walking Condition* and *Task Condition* for perceived mental workload [*F*_(1, 19)_ = 0.97, *p* = 0.337, $\eta_{p}^{2}$ = 0.05] and physical workload [*F*_(1, 19)_ = 1.21, *p* = 0.285, $\eta_{p}^{2}$ = 0.06]. However, there were significant main effects of *Walking Condition* on mental workload [*F*_(1, 19)_ = 44.58, *p* < 0.001, $\eta_{p}^{2}$ = 0.70] and physical workload [*F*_(1, 19)_ = 27.56, *p* < 0.001, $\eta_{p}^{2}$ = 0.59], indicating an increase of perceived mental and physical workload during exoskeleton-assisted walking compared to unassisted walking. *Task Condition* had a significant effect on mental workload [*F*_(1, 19)_ = 79.77, *p* < 0.001, $\eta_{p}^{2}$ = 0.81], but no effect on physical workload [*F*_(1, 19)_= 0.01, *p* = 0.914, $\eta_{p}^{2}$ = 0.00]. This shows that the subtraction task in the dual-task condition increased mental workload as intended, without affecting physical workload.

The Friedman test revealed significant effects of *Time Condition* on perceived mental workload [$\chi_{\left(2\right)}^{2}$ = 17.10, *p* < 0.001] and physical workload [$\chi_{\left(2\right)}^{2}$ = 9.10, *p* = 0.011]. Dunn-Bonferroni *post-hoc* tests indicated that both mental workload (*p* < 0.001) and physical workload (*p* = 0.027) decreased significantly from the first to the third trial. The differences between the first and the second trial were not significant for mental workload (*p* = 0.342) and physical workload (*p* = 0.707). Similarly, no significant differences were found between the second and the third trial for mental workload (*p* = 0.066) and physical workload (*p* = 0.464).

## 4 Discussion

This study employed a dual-task paradigm to investigate cognitive-motor interference (H1) and short-term familiarization effects (H2) in an outdoor walking experiment with a powered lower-limb exoskeleton. In contrast to unassisted walking, introducing the secondary task during exoskeleton-assisted walking slightly increased gait velocity and decreased stride time variability. Concurrently, cognitive performance declined during exoskeleton-assisted walking, suggesting a shift to a *posture-first* strategy. Short-term familiarization was observed through reduced perceived workload and improved cognitive performance throughout the session. However, cognitive performance remained inferior to the seated control condition and unassisted walking, suggesting that walking with the exoskeleton still requires significant attentional resources.

### 4.1 Introduction of a cognitive task increased gait velocity and decreased stride time variability in exoskeleton-assisted walking

Previous studies have shown that gait velocity decreases under dual-task conditions, suggesting that higher-order cognitive processes are involved (Al-Yahya et al., 2011). In line with this, the present study found a significant decrease in gait velocity during unassisted walking with a subtraction task (−9%). However, gait velocity increased during dual-task exoskeleton-assisted walking (+22%), suggesting that, despite the mechanical constraints imposed by the TWIN, gait patterns were modified—possibly through increased stride length. In a self-paced treadmill walking study by Gupta et al. (2023), healthy adults exhibited a tendency to shift toward a preferred walking velocity while performing a secondary task, suggesting that walking at a preferred velocity may require less attentional effort in dual-task scenarios. It can therefore be hypothesized that participants walking at low speeds with the exoskeleton exhibit similar behavior.

Similarly, stride time variability increased from single- to dual-task conditions during unassisted walking (+83%) but slightly decreased during exoskeleton-assisted walking (−12%), contradicting the initial hypothesis that motor interference increases during exoskeleton-assisted walking. Stride time variability was significantly higher during exoskeleton-assisted walking compared to unassisted walking in single-task conditions, but no differences emerged in the dual-task condition. The introduction of a secondary task appeared to enhance motor performance during exoskeleton-assisted walking, consistent with participants' feedback that it shifted their attention away from the exoskeleton. This shift in attention may have reduced exploratory behavior compared to single-task walking, potentially decreasing resistance against the exoskeleton and promoting more automatic compliance with the predefined gait trajectories.

Treadmill studies have shown that externalizing the focus of attention in this way can reduce gait variability during simple secondary tasks, as it promotes more automatic gait execution (Decker et al., 2016; Lövdén et al., 2008; Riedel et al., 2024). Such findings have not been replicated for overground walking, raising questions about their transferability (Hybart and Ferris, 2023; Wrightson et al., 2020). However, similar to a treadmill, the TWIN induces a rhythmic regulating mechanism through predefined trajectories, haptic cues via the transmission of forces and torques (Wu et al., 2022), and auditory cues as the device beeps before the initiation of each step. There is evidence that rhythmic auditory stimulation can lead to increased gait speed and stride length, as well as reduced gait variability in individuals with Parkinson's disease (Hausdorff et al., 2007; Pau et al., 2016). Moreover, studies with lower-limb exoskeletons show that external cues can improve motor performance (Kim et al., 2022; Wu et al., 2022). From an ergonomic perspective, the results suggest that providing direct feedback through external cues may benefit exoskeleton users—particularly when their attention is externally directed, as often occurs in real-world contexts.

### 4.2 Differing prioritization strategies for unassisted walking and exoskeleton-assisted walking

Reduced gait velocity and increased stride time variability during unassisted walking suggest that participants prioritized the cognitive task over the motor task, employing a *posture-second* strategy (Yogev-Seligmann et al., 2012). This is supported by the results of cognitive performance, as correct response rates (CRR) did not differ significantly from the seated control condition. In contrast, CRR significantly decreased during exoskeleton-assisted walking (−14%), indicating that fewer cognitive resources were allocated to the secondary task. This suggests a shift to a *posture-first* strategy. Conversely, participant feedback indicated that the dual-task condition led to more automated walking, as they did not consciously think about walking, suggesting that a *posture-second* strategy may also be present during exoskeleton-assisted walking. However, walking with the exoskeleton and coordinating with external cues still required considerable attentional resources, contributing to the observed decline in cognitive performance. This implies that only a portion of attentional resources was allocated to the secondary task, with the primary focus remaining on the motor task. This shift toward a *posture-first* strategy implies that participants perceived exoskeleton-assisted walking as a postural threat, which is consistent with the fact that the TWIN restricts natural movement. This underscores the need for enhancing the intuitiveness of the exoskeleton's operation through the incorporation of adaptive control systems (Baud et al., 2021) and mechanical designs that permit natural joint motion without limiting degrees of freedom (Dežman et al., 2024).

In human–robot interaction research, particularly in non-physically coupled systems, the ability of a robot to anticipate human behavior has been shown to improve efficiency (Huang and Mutlu, 2016). A key challenge involves the integration of intent recognition that infer user intent and dynamically adapt the level of exoskeletal assistance. The effectiveness of these mechanisms is closely tied to the concept of mental models, the user's internal understanding of how the exoskeleton functions (Stirling et al., 2019). Discrepancies between the user's mental model and the system's embedded model can result in reduced performance. Empirical evidence indicates that both the type of the control strategy (Zhang et al., 2015) and the temporal dynamics of assistance (Ding et al., 2016) are critical determinants of user performance, potentially due to their varying degrees of congruence with the user's mental model. Future investigations should systematically examine which adaptive control strategies best match user's mental models under various task demands.

Participants in this study reported significantly higher physical and mental workload during exoskeleton-assisted walking compared to unassisted walking, suggesting that the exoskeleton imposes greater demands on both cognitive and motor control systems. While cognitive performance declined, motor performance improved. These findings indicate that, although external rhythmic cues can enhance motor performance, walking with the exoskeleton and coordinating with these cues still require substantial attentional resources. The integration of multimodal feedback systems, including visual, auditory, and haptic cues that are easy to interpret, may help reduce cognitive workload during exoskeleton use (Wu et al., 2022) and warrants further investigation in future studies.

### 4.3 Short-term familiarization effects in exoskeleton-assisted walking

To test the second hypothesis regarding short-term, within-session familiarization effects during exoskeleton-assisted walking, the dependent variables were assessed across three consecutive single-task trials (see Figure 2). Each exoskeleton trial lasted ~3–4 min, with the entire session spanning around 20 min. Participants reported a significant reduction in both perceived physical and mental workload throughout the session, indicating familiarization. These results align with those of Marinou et al. (2022), who found that participants began exhibiting familiarization effects with the TWIN exoskeleton within 10–20 min of walking.

In addition, the cognitive performance increased significantly from the first to the third trial, supporting the hypothesis that greater alignment with the exoskeleton's assistance reduces cognitive load. However, cognitive performance remained inferior to both seated control and unassisted walking conditions, suggesting that walking with the exoskeleton still requires significant attentional resources. The dependent variables showed no significant differences between the second and third trials, indicating that familiarization effects plateaued after the second trial. However, long-term study designs are required to ascertain whether familiarization has been fully completed or not. The time required for familiarization can vary depending on several factors, including the complexity of the exoskeleton (e.g., the number of assisted joints and the magnitude of assistance) and the individual characteristics of the user (Poggensee and Collins, 2021). In line with the recommendations of Poggensee and Collins (2021), designs of future experiments investigating human-exoskeleton interaction should ensure that participants are thoroughly familiarized.

### 4.4 Limitations

The participants in this study did not represent the intended target user group for the TWIN exoskeleton, which intentionally interfered with natural walking. The use of healthy young adults as participants limits the generalizability of the results, as cognitive-motor challenges may differ in clinical populations. While assessing the efficacy of a specific exoskeleton ideally necessitates the recruitment of individuals from the target user group, such an objective was beyond the scope of the current investigation. Future research should include individuals with spinal cord injury or other relevant clinical populations to enhance the ecological validity of the results. All participants were novices with the TWIN exoskeleton. Although the study controlled for familiarization effects before examining cognitive-motor interference, the results do not allow for a definitive conclusion regarding whether the familiarization process was fully completed, highlighting the need for a longer familiarization period. Additionally, participants had to coordinate their movements with both the exoskeleton and crutches, thereby introducing additional cognitive and motor demands. Another important factor to consider is the substantial difference in gait velocity between unassisted and exoskeleton-assisted walking, as gait velocity influences gait variability. Jordan et al. (2007) provide evidence that gait variability tends to decrease as walking speed increases. The observed differences in motor performance in response to the secondary task can be interpreted independently of gait velocity within each walking condition. However, the overall increase in gait variability during exoskeleton-assisted walking as compared to unassisted walking may be due to the slower walking speed rather than being attributable solely to the use of the exoskeleton.

Additionally, the subtraction task had a predictive rhythmic pattern, which may have led participants to synchronize their response speed with the exoskeleton's gait cycle, despite instructions to perform the task as quickly and accurately as possible (de Bartolo et al., 2021). Exploratory *t*-tests revealed a significant reduction in response rate from unassisted walking (0.36 responses/s) to exoskeleton-assisted walking (0.31 responses/s), while accuracy remained relatively unchanged (unassisted walking: 97.69%; exoskeleton-assisted walking: 96.78%). Using unpredictable stimuli may help address this potential confounding factor. For example, Reiser et al. (2022) jittered the stimulus interval by ±250 ms. In outdoor experiments, responding to jittered auditory stimuli may provide a viable alternative. Finally, randomizing the initial number on each trial in the subtraction task may have introduced variability in task complexity across trials. This approach was intended to reduce predictability and reduce the potential for rehearsal effects.

## 5 Conclusion

This study employed a dual-task paradigm to investigate cognitive-motor interference and short-term familiarization effects in an outdoor walking experiment with healthy adults using the TWIN lower-limb exoskeleton. The findings indicate the adoption of different prioritization strategies for unassisted and exoskeleton-assisted walking. Specifically, introducing a secondary task during exoskeleton-assisted walking slightly increased gait velocity and decreased stride time variability, possibly due to the partial allocation of attentional resources enhancing coordination with the exoskeleton's external rhythmic cues. However, cognitive performance declined compared to both the seated control and unassisted walking conditions, indicating a *posture-first* strategy. Short-term familiarization during exoskeleton-assisted walking reduced perceived workload and improved cognitive performance. Nonetheless, performance remained below that of control conditions, suggesting that walking with the exoskeleton still requires significant attentional resources. These findings should be interpreted with caution, given the limited familiarization period and the exclusive focus on healthy young adults. Further research is needed to validate these preliminary results across larger and more diverse populations, and to examine the effects of extended training periods. Future studies should also explore adaptive control and user feedback systems to enhance the efficiency of human-exoskeleton interaction. Ultimately, the results underscore the importance of designing exoskeletons with a good cognitive fit to support the perception-cognition-action decision process of the exoskeleton user (Stirling et al., 2020).

## Data Availability

The raw data supporting the conclusions of this article will be made available by the authors, without undue reservation.
